# Health Outcomes of Sarcopenia: A Systematic Review and Meta-Analysis

**DOI:** 10.1371/journal.pone.0169548

**Published:** 2017-01-17

**Authors:** Charlotte Beaudart, Myriam Zaaria, Françoise Pasleau, Jean-Yves Reginster, Olivier Bruyère

**Affiliations:** 1 Public Health, Epidemiology and Health Economics, University of Liège, Liège, Belgium; 2 Aix-Marseille University, School of Medicine, Marseille, France; 3 Life Sciences Library, University of Liège, Liège, Belgium; University of British Columbia, CANADA

## Abstract

**Objective:**

The purpose of this study was to perform a systematic review to assess the short-, middle- and long-term consequences of sarcopenia.

**Methods:**

Prospective studies assessing the consequences of sarcopenia were searched across different electronic databases (MEDLINE, EMBASE, EBM Reviews, Cochrane Database of Systematic Reviews, EBM Reviews ACP Journal Club, EBM Reviews DARE and AMED). Only studies that used the definition of the European Working Group on Sarcopenia in Older People to diagnose sarcopenia were included. Study selection and data extraction were performed by two independent reviewers. For outcomes reported by three or more studies, a meta-analysis was performed. The study results are expressed as odds ratios (OR) with 95% CI.

**Results:**

Of the 772 references identified through the database search, 17 were included in this systematic review. The number of participants in the included studies ranged from 99 to 6658, and the duration of follow-up varied from 3 months to 9.8 years. Eleven out of 12 studies assessed the impact of sarcopenia on mortality. The results showed a higher rate of mortality among sarcopenic subjects (pooled OR of 3.596 (95% CI 2.96–4.37)). The effect was higher in people aged 79 years or older compared with younger subjects (p = 0.02). Sarcopenia is also associated with functional decline (pooled OR of 6 studies 3.03 (95% CI 1.80–5.12)), a higher rate of falls (2/2 studies found a significant association) and a higher incidence of hospitalizations (1/1 study). The impact of sarcopenia on the incidence of fractures and the length of hospital stay was less clear (only 1/2 studies showed an association for both outcomes).

**Conclusion:**

Sarcopenia is associated with several harmful outcomes, making this geriatric syndrome a real public health burden.

## Introduction

The aging process is responsible of many changes in body composition including a loss of skeletal muscle mass. From the age of 25, there is a progressive decrease in the size and number of muscle fibers resulting in a total decrease of about 40% in muscle mass between the ages of 25 and 80 years[1]. Beyond some defined threshold, this age-related loss of muscle mass is characterized as abnormal. To define this a progressive loss of muscle mass with advancing age the term sarcopenia was first coined by Rosenberg et al. in 1989[2]. This very first definition included only the notion of muscle mass. However, with time, the definition has expanded to incorporate the notion of muscle function, including reduced muscle strength and/or physical performance. Indeed, several epidemiological studies showed a decline in muscle strength 2–5 times greater than the decline in muscle mass over the same period of time[3,4]. Although muscle mass is a determinant of muscle strength, the loss in muscle mass with advancing age is far from the sole or even primary explanation for the loss of muscle strength. Furthermore, maintenance or gain in muscle mass does not necessarily prevent age-related decline in muscle strength[5]. This dissociation between the loss in muscle mass and the loss in muscle strength can partly be explained by the atrophy and denervation of the muscle fibers. In addition, neural changes, such as a decline in motor unit recruitment and in motor unit discharge rates, also contribute to this dissociation[6].

Currently, several definitions of sarcopenia have been proposed[7–15], but no worldwide consensus has yet been reached. It is important to note that sarcopenia is now recognized as an independent condition by an ICD-10-CM code[16].

Currently, some potential consequences of sarcopenia on individual health and public health[17] have been suggested, including physical disabilities, depression, decreased quality of life, nursing home admission and even death. However, it is not always clear whether these consequences were determined from longitudinal studies or simply from cross-sectional studies, in which case it would be incorrect to define these health issues as “consequences”; they would be more appropriately called “associations”. Moreover, it appears that the consequences of sarcopenia can vary according to the operational definition used for the diagnosis of sarcopenia. For example, Bishoff-Ferrari[18] compared the ability of different operational definitions to predict falls. It appears that the relative risk (RR) of falls for sarcopenia patients could vary from 1.82 (95% CI 1.24–2.69) to 0.61 (95% CI 0.24–1.55) depending on the definition used to diagnose sarcopenia.

To avoid ambiguity surrounding the interpretation of the consequences of sarcopenia and move gradually, it would be interesting to identify the consequences of sarcopenia related to one unique definition of sarcopenia. A couple of years ago, the European Working Group on Sarcopenia in Older People[8] reached a consensus and defined sarcopenia as a progressive and generalized loss of muscle mass and muscle function (muscle strength or physical performance) with advancing age. To reinforce its validity, this recent operational definition still needs to show its ability to predict the clinical outcomes of sarcopenia.

The aim of this research is therefore to identify all short-, middle- and long-term consequences of sarcopenia, as defined by the European Working Group on Sarcopenia in Older People (EWGSOP)[8], specifically reported in prospective studies.

## Methods

The Preferred Reporting Items for Systematic Reviews and Meta-analysis (PRISMA) statement (S1 Table) has been followed for all steps of this research.

### Literature search

The electronic databases MEDLINE, EMBASE, Cochrane Database of Systematic Review, ACP Journal Club, Database of Abstracts of Reviews of Effects (DARE) and Allied and Complementary Medicine (AMED) were searched for cohort studies assessing the clinical and health consequences of sarcopenia. No date limit was applied. The search strategy and search terms used for this research are detailed in Table 1. Additional studies were identified through a manual search of the bibliographic references of relevant articles and existing reviews.

**Table 1:** Search strategy.

1. Sarcopenia/
2. Sarcopeni$.tw
3. Ewgsop.tw
4. Exp cohort studies/
5. Cohort stud$.tw
6. Cohort analy$.tw
7. Longitudinal stud$.tw
8. Prospective stud$.tw
9. Observational stud$.tw
10. Or/1-3
11. Or/4-10
12. And/11-12

### Study selection

In the initial screening stage, two investigators independently reviewed the title and abstract of each of these references to exclude articles irrelevant to the systematic review. Rigorous inclusion criteria were adhered to (Table 2). In the second step, the two investigators independently read the full texts of the articles that were not excluded in the initial stage, then selected the studies that met the inclusion criteria. All differences of opinion regarding the selection of articles were resolved through discussion and consensus.

**Table 2:** Inclusion criteria.

Design	Prospective studies (with at least two prospective evaluations)
Participants	Human, middle-aged and elderly men and women
Diagnosis of sarcopenia	Based on the EWGSOP definition (presence of low muscle mass + either low muscle strength or low physical performance (low gait speed or low SPPB test)).
Outcome	Report of at least one outcome of sarcopenia
Language	English

Studies dealing with sarcopenia associated with cancer cachexia or neurological diseases, any malignant disease, inflammatory or autoimmune diseases, corticosteroids for systemic use and obesity were excluded.

### Data extraction

Data were extracted independently by two reviewers according to a standardized data extraction form. The following data were extracted: authors; journal name; year of publication; country; objective of the study; socio-demographic data (country, type of population, sex ratio, mean age); sample size; design (length of intervention, number of groups, description of groups); tools used to assess muscle mass, muscle strength and physical performance; reported prevalence of sarcopenia; outcomes; conclusion; presence of conflicts of interest; and potential funding.

To include as many studies as possible in our systematic review, we systematically contacted authors or co-authors when information was missing in the full-text paper.

### Methodology quality assessment

The assessment of methodological quality was performed independently by two reviewers using the Newcastle-Ottawa Scale (NOS). A quality score was calculated based on three categories: group selection (four items), comparability between groups (one item), and outcome and exposure assessment (3 items). A maximum of one star could be awarded for each item in the group selection and outcome and exposure assessment categories. A maximum of two stars could be awarded for comparability. Thus, the maximum possible score was nine stars, which represented the highest methodological quality. Studies were considered high quality if they scored above the median of eight stars. Disagreements between the reviewers were discussed until consensus was reached.

### Synthesis of the results

The findings were evaluated in a descriptive manner based on the information provided by each of the included studies. For outcomes reported by three or more studies, a meta-analysis was performed. Study results were expressed as odds ratios (OR) with 95% CI. When available, adjusted ORs were reported. Otherwise, crude ORs were computed from the available results in the paper. We decided to use/compute ORs instead of HRs because HRs were not available for all studies and were impossible to compute with the data available in the different papers. When one study reported results for different time points, we decided to include only the results for the longest follow-up point. To evaluate the impact of individual studies on the overall results, we performed a one-way sensitivity analysis by omitting one study at a time and then repeating the analysis.

Since participant demographics and clinical settings differed among studies, we assumed the presence of heterogeneity a priori. Therefore, we used a random-effects model to pool the results. We assessed heterogeneity using the χ_2_-test of heterogeneity and the *I*^2^ measure of inconsistency. Moreover, analyses of subgroups based on the clinical setting (community-dwelling, hospitalized and institutionalized people), NOS score (according to the median quality), age (according to the median age), length of follow-up (according to the median length of follow-up) and the tool used to measure muscle mass (Dual-Energy X-Ray Absorptiometry (DXA), Bioelectrical impedance analysis (BIA) or anthropometric measurements) were performed. A test of interaction using a mixed-effects model was performed for all subgroups to establish whether the difference in effect size among subgroups was statistically significant.

Potential publication bias was explored by means of a funnel plot. We used the Egger’s regression asymmetry test to detect publication bias.

For all results, a two-sided p value of 0.05 or less was considered significant. All analyses were performed using the software package Comprehensive Meta Analysis, Biostat v2.

## Results

### Search strategy

**Fig 1:**
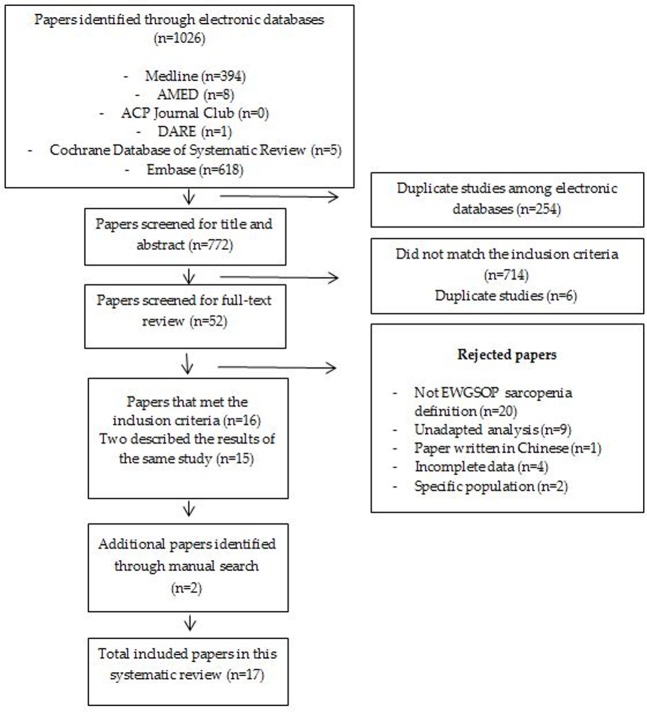
Search strategy.

A total of 1026 studies were identified through electronic database searches. Among these studies, we were able to remove 254 duplicates. Therefore, 772 articles were screened for title and abstract by two independent reviewers. Only 16 studies met the inclusion criteria, but two of these studies described the same results. As a result of a manual search of the bibliographies of pertinent papers, we were able to identify two additional studies. Therefore, 17 prospective studies assessing the outcomes of sarcopenia, defined according to the EWGSOP guidelines, were included in this systematic review (Fig 1).

### Included studies

All of these studies (characteristics presented in Table 3) were quite recent since they were published between 2012 and 2015. Most of the studies were performed in Europe (9/17 studies[19–27]), while 5 were performed in America (2 in USA, 3 in South America) [28–32], and 3 were performed in Asia[33–35]. All of the studies included subjects aged 60 years or older; 11 studies included community-dwelling older people[21,22,25,29–35], 4 included hospitalized subjects[19,20,24,27], and 2 involved nursing home residents[23,26]. Only one study included only men[32]; all of the others were mixed-gender studies, with the percentage of women varying between 48.9%[35] and 75%[23]. The number of participants ranged from 99[20] to 6658[31], and the duration of follow-up varied from 3 months[20,24,27] to 9.8 years[32]. Sarcopenia was diagnosed according to the algorithm proposed by the EWGSOP. Muscle mass was measured using bioelectrical impedance analysis (BIA) in the majority of studies (7/17 studies[19,20,23–25,27,33]), followed by anthropometric measurements (6/17 studies[21,22,26,28–30]) and, finally, by Dual-Energy X-ray Absorptiometry (DXA) (4/17 studies[31,32,34,35]). Muscle strength was measured using handgrip strength in all of studies except for one[35], which used an isokinetic device. Finally, only one study used the Short Physical Performance Battery (SPPB)[35] test to measure physical performance; all other studies used gait speed. The prevalence of sarcopenia varied from 4.3% in a population of ambulatory community-dwelling men[32] to 73.3% among nursing home residents in Turkey[26]. The majority of studies declared their source of funding (funding from a local foundation[28,29], from a national ministry[19,33], from a grant[21,22] and from a national institute of research[25,31,32,35]) if any (two studies[20,26] declared no funding). Five other studies did not report the presence or absence of funding[23,24,27,30,34]. Eleven studies reported no conflict of interest[19–24,26,27,33–35], 2 reported one conflict of interest[31,32] and 4 studies did not report this information[28,29].

**Table 3:** Study characteristics.

First author's name	Sociodemographic data (Country, type of population, mean age, sex ratio)	Sample size	Design (time of follow up, number of groups, description of groups)	Tool used to assess muscle mass	Tool used to assess muscle strength	Tool used to assess physical performance	Prevalence of sarcopenia	Outcomes
da Silva, 2014a[28]	Brazil, Community-dwelling adults, Age ≥ 60 years, Age: 69.6 ± 0.6 years, Women: 59.5%	1149	5 years (mean follow-up of 4.19 ± 0.4 years), Two groups: Sarcopenic / Non-sarcopenic	Anthropometric equation: Lee equation	Hand-held dynamometer	Gait speed determined by the walk test of the SPPB (4 m)	15.4%	Mortality
da Silva, 2014b[29]	Brazil, Community-dwelling adults, Age ≥ 60 years, Age: 68.9 ± 0.4 years, Women: 56.5%	328	4 years, Two groups: Sarcopenic / Non-sarcopenic	Anthropometric equation: Lee equation	Hand-held dynamometer	Gait speed determined by the walk test of the SPPB (4 m)	13.4%	Functional disability
Vetrano, 2014[19]	Italy, Hospitalized patients, Age ≥ 65 years, Age: 80.8 ± 7 years, Women: 56%	In-hospital mortality: 770 / 1-year mortality: 650	1 year, Two groups: Sarcopenic / Non-sarcopenic	BIA	Hand-held dynamometer	Gait speed (4 m)	28%	Mortality (in hospital, one-year mortality)
Sanchez-Rodriguez, 2014[20]	Spain, Hospitalized patients, Age ≥ 75 years, Age: 84.6 ± 6.6 years (range: 76 to 80.5 years), Women: 61,6%	99	3 months, Two groups: Sarcopenic / Non-sarcopenic	BIA	Hand-held dynamometer	None	46.5%	Mortality, Functional status
Sánchez-Rodríguez, 2015[27]	Spain, Hospitalized patients, Age ≥ 70, Age: 84,1 ± 8,5 years, Women: 62 (62%)	100	3 months, Two groups: Sarcopenic / Non-sarcopenic	BIA	Hand-held dynamometer	Gait speed (4 m)	58%	Functional status, Length of stay
Tanimoto, 2013[33]	Japan, Community-dwelling elderly, Age: ≥ 65, Age: Men: 73.3 ± 5.9 years / Women: 73.1 ± 6.2 years, Women: 63,4% (471)	716	2 years Three groups: Sarcopenic / Intermediate / Non-sarcopenic	BIA	Hand-held dynamometer	Gait speed (5 m)	9.36%	Functional disability
Arango-Lopera, 2013[30]	Mexico, Community-dwelling elderly, Age: ≥ 70, Age: 78.5 ±7 years, Women: 53.3%	345	3 years, Two groups: Sarcopenic / Non-sarcopenic	Calf circumference	Hand-held dynamometer	Gait speed	33.6%	Mortality
Landi, 2013[21]	Italy, Frail octogenarians living in the community, Age: 80 to 85 years, Age: 82.2 ± 1.4 years, Women: 131 (66.5%)	197	7 years, Two groups: Sarcopenic / Non-sarcopenic	Mid-arm muscle circumference (MAMC)	Handgrip dynamometer	Gait speed (4 m)	21.8%	Mortality
Landi, 2012a[22]	Italy, Community-dwelling individuals, Age: ≥ 80, Age: 86,7 ± 5.4 years, Women: 177 (68%)	260	2 years, Two groups: Sarcopenic / Non-sarcopenic	Mid-arm muscle circumference (MAMC)	Handgrip dynamometer	Gait speed (4 m)	25.4%	Fall
Landi, 2012b[23]	Italy, Elderly adults living in a nursing home, Age: ≥ 70, Age: 84.1 ± 4.8 years, Women: 91 (75%)	122	6 months, Two groups: Sarcopenic / Non-sarcopenic	BIA	Handgrip dynamometer	Gait speed (4 m)	32.8%	Mortality
Cerri, 2015[24]	Italy, Elderly adults hospitalized with malnutrition or at risk of malnutrition, Age: ≥ 65 years, Age: (years) 84.2 ± 7.1 (range: 66–100), Women: 61 (59.2%)	103	3 months, Three groups: Sarcopenic / Non-sarcopenic / Uncertain diagnosis	BIA	Handgrip dynamometer	Gait speed (4 m)	21.4%	Mortality
Woo, 2015[34]	China, Community-living elderly adults, Age: ≥ 65 years, Mean age: 75,4 years, Women: 246 (55.2%)	Varying between 1872 and 4000, depending on the outcome	4–10 years, depending on the outcome of interest, Two groups: Sarcopenic / Non-sarcopenic	DXA	Handgrip dynamometer	Gait speed (6 m), Chair stands	9.02%	Mortality, Functional disability, Length of stay
Bianchi, 2015[25]	Italy, Community-dwelling elderly adults, Age: ≥ 65 years, Age: 77.1 ± 5.5, Women: 288 (53.5%)	538	55 months (median of follow-up), Three groups: Sarcopenic / Pre-sarcopenic / Non-sarcopenic	BIA	Handheld dynamometer	Gait speed (4 m)	10.2%	Mortality, Hospitalization, Functional disability
Chalhoub, 2015[31]	USA, Community-living elderly adults, Age: ≥ 65 years, Mean age: 76.8 years, Women: 16.7% (1114), Men: 5544	6658	Men (MrOS): 9 years, Women (SOF): 8 years, 4 groups:Normal BMD, No sarcopenia / Normal BMD, Sarcopenia / Low BMD, No sarcopenia /Low BMD, Sarcopenia	DXA	Dynamometer	Gait speed (6 m)	5.57%	Fractures
Saka, 2015[26]	Turkey, Nursing home residents, Age: ≥ 65 years, Mean age: 78.0 ± 7.9 years (65–101), Women: 49% (199)	402	1 year, 4 groups: Sarcopenia—MN/MR / Sarcopenia—MN/MR + / Sarcopenia + MN/MR / Sarcopenia + MN/MR +	Anthropometric measurements: Calf circumference, Mid-upper arm circumference	Handheld dynamometer	Gait speed (4 m)	73.3%	Mortality
Cawthon, 2015[32]	USA, Ambulatory community-dwelling men, Age: ≥ 65 years, Mean age: 76.6 years, 100% men	Varying between 3726 and 5934, depending on the outcome	9.8 years, Two groups: Sarcopenic / Non-sarcopenic	DXA	Handgrip strength	- Gait speed (6 m), Average of two trials, Chair stands	4.3%	Mortality, Falls, Fractures, Functional limitations
Kim, 2014[35]	Korea, Community-dwelling older adults, Age: ≥ 65 years, Mean age: 73.6 years, Women: 48.9% (272)	556	6 years, Two groups: Sarcopenic / Non-sarcopenic	DXA	Isokinetic device at an angular velocity of 60°/s	SPPB score	ASM/ht2: 8.8%, ASM/wt: 26%	Mortality

The studies reported results for approximately 6 different types of consequences: mortality (12 studies[19–21,23–26,28,30,32,34,35]), functional decline (7 studies[20,25,27,29,32–34]), falls (2 studies[22,32]), fracture (2 studies[31,32]), length of hospital stay (2 studies[27,34]) and hospitalization (1 study[25]).

### Mortality

**Fig 2:**
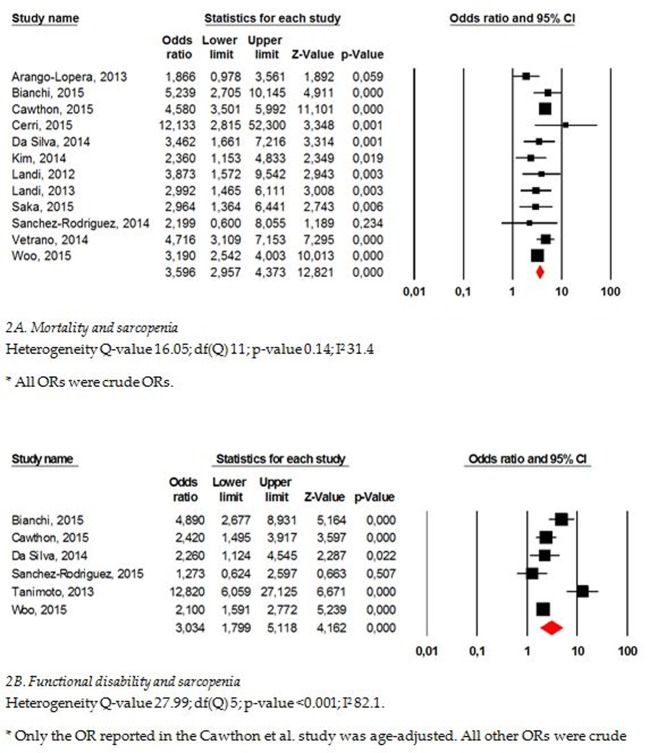
Mortality (A) and functional decline (B) as outcomes of sarcopenia.

A total of 12 studies reported results for mortality[19–21,23,25,26,28,30,32,34,35]. One study scored 5/9[20] on the NOS, 4 scored 7/9[19,24,32,34], 4 scored 8/9[21,25,30,35] and 3 scored 9/9[23,26,28], indicating excellent quality (Table 4). The population was composed of ambulatory community-dwelling subjects in 7 of these studies[21,25,28,30,32,34,35], hospitalized subjects in 3 studies[19,20,24] and nursing home residents[23,26] in the two last. A higher risk of mortality was found for sarcopenic subjects compared with non-sarcopenic ones in 10/12 studies. In one[36] of these studies, the results were only significant for sarcopenic men and not for sarcopenic women. A meta-analysis was performed to compute the results of these different studies. Because we contacted the authors or co-authors when information was missing from the full-text paper, we were able to obtain the ORs of all studies. An overall OR of 3.596 (95% CI 2.96–4.37) was found, indicating a higher risk of mortality for sarcopenic subjects compared with non-sarcopenic ones (Fig 2A). Egger’s regression analysis showed that publication bias was not present (p = 0.80). The results of the subgroup analyses are available in Table 5. A significant difference in effect was found only for age, with a significantly higher association between sarcopenia and mortality in subjects aged 79 years or older (OR 4.42 (95% CI 3.60–5.42)) compared with younger subjects (OR 3.09 (2.49–3.84); p = 0.02).

**Table 4:** NOS scores.

Study	Selection (4 stars)	Comparability (2 stars)	Exposure (3 stars)	Total score (9 stars)
da Silva, 2014a[28]	4 stars	2 stars	3 stars	9 stars
da Silva, 2014b[29]	4 stars	2 stars	2 stars	8 stars
Vetrano, 2014[19]	3 stars	1 star	3 stars	7 stars
Sanchez-Rodriguez, 2014[20]	3 stars	0 star	2 stars	5 stars
Sánchez-Rodríguez, 2015[27]	3 stars	0 star	3 stars	6 stars
Tanimoto, 2013[33]	3 stars	2 stars	3 stars	8 stars
Arango-Lopera, 2013[30]	4 stars	2 stars	2 stars	8 stars
Landi, 2013[21]	4 stars	2 stars	2 stars	8 stars
Landi, 2012a[22]	4 stars	2 stars	2 stars	8 stars
Landi, 2012b[23]	4 stars	2 stars	3 stars	9 stars
Cerri, 2015[24]	3 stars	2 stars	2 stars	7 stars
Woo, 2015[34]	3 stars	2 stars	2 stars	7 stars
Bianchi, 2015[25]	4 stars	2 stars	2 stars	8 stars
Chalhoub, 2015[31]	4 stars	2 stars	1 star	7 stars
Saka, 2015[26]	4 stars	2 stars	3 stars	9 stars
Cawthon, 2015[32]	4 stars	2 stars	1 star	7 stars
Kim, 2014[35]	4 stars	2 stars	2 stars	8 stars

**Table 5:** Subgroup analyses.

	Number of studies	OR (95% CI)	p-value
**Mortality**			
Clinical settings			
*Community*	7	3.39 (2.65–4.33)	0.63
*Hospital*	3	4.73 (2.46–9.12)	
*Nursing home*	2	3.32 (1.84–5.98)	
Age			
*≤ 79 years*	6	3.09 (2.49–3.84)	0.02
*> 79 years*	6	4.42 (3.60–5.42)	
Length of follow-up			
*≤ 36 months*	6	3.31 (2.17–5.07)	0.23
*> 36 months*	6	3.72 (3.02–4.60)	
NOS score			
*≤ 7 points*	5	4.06 (3.06–5.38)	0.16
*> 7 points*	7	3.05 (2.32–4.01)	
Tool used for muscle mass measure			
*BIA*	4	4.84 (3.47–6.74)	0.06
*DXA*	4	3.58 (2.73–4.63)	
*Anthropometric measures*	4	2.67 (1.84–3.87)	
**Functional decline**			
Age			
*≤ 75 years*	3	3.79 (1.36 -10-6)	0.52
*> 75 years*	3	2.52 (1.26–5.03)	
Length of follow-up			
*≤ 51*.*5 months*	3	3.31 (0.87–12.55)	0.79
*> 51*.*5 months*	3	2.75 (1.75–4.31)	
Tool used for muscle mass measure			
*BIA*	3	4.24 (2.87–6.27)	0.29
*DXA*	3	2.18 (1.74–2.74)	

Nb. Subgroup analyses for clinical settings and NOS score could not be performed for functional decline given the limited number of studies for these groups (one unique study with a NOS score ≤ 7 and one unique study performed with hospitalized subjects).

### Functional decline

Seven studies[20,25,27,29,32–34] reported results regarding the association between sarcopenia and the incidence of functional disability. However, two individual studies[20,27] reported similar results for a similar population for the outcome of functional decline. We decided to keep the most recent study in our analysis. Therefore, only 6 studies were analyzed for this outcome (1 with an NOS score of 6/9[27], 2 with an NOS score of 7/9[32,34], 3 with an NOS score of 8/9[25,29,33]). Five out of these 6 studies found a significantly greater decline of function (assessed using the ADL-Katz scale[25,29,33], the IAD-Lawton scale[25,29,33], the Barthel Index[27] and self-reported functional limitations[32,34]) in sarcopenic subjects compared with non-sarcopenic subjects. However, in one of these studies[34], the association was significant only for men and not for women. The pooled results indicated a higher risk of functional disability for sarcopenic subjects compared with non-sarcopenic ones (pooled OR 3.03 (95% CI 1.80–5.12)). No publication bias was found for this meta-analysis (p = 0.37). The results of the subgroup analyses are available in Table 5. No effect of age, length of follow-up or of tool used to measure muscle mass was found.

### Falls

Two studies[22,32] (one with a score of 7/9 on the NOS[32], one with a score of 8/9[22]) reported results for the association between sarcopenia and the incidence of falls. One of these studies was performed on 260 community-dwelling individuals with a mean age of 86.7±5.4 years who were followed for 2 years to determine the incidence of falls[22]. The second study was performed on 5828 ambulatory community-dwelling individuals for whom the incidence of falls was recorded 3 times per year for 1 year. Both studies found a significant association between sarcopenia and the incidence of falls. In the first study[22], 27.3% of the sarcopenic subjects fell at least one time, compared with 9.8% of the non-sarcopenic ones (p<0.001). A crude HR of 3.45 (95% CI 1.68–7.09) was reported. The HR was still significant in a fully adjusted model (adjusted for age, gender, cognitive impairment, ADL impairment, sensory impairments, body mass index, depression, physical activity, cholesterol, stroke, diabetes, number of medications, and reactive C protein) that resulted in an HR of 3.23 (95% CI 1.25–8.29). In the second study[32], the authors found a higher risk of recurrent falls (at least 2 falls in one year) for sarcopenic subjects, with a significant OR equal to 2.38 (95%CI 1.75–3.23) when adjusted for age.

### Fractures

Two studies[31,32] followed sarcopenic subjects to assess the incidence of fractures. The first study[31], which had an NOS score of 7/9, followed 5544 elderly men and 1114 women living in the community for 9 years and 8 years, respectively. The studies defined 4 groups: subjects with normal bone mineral density (BMD) and no sarcopenia, subjects with normal BMD but with sarcopenia, subjects with low BMD but no sarcopenia and, finally, subjects with low BMD and sarcopenia. The authors found a significantly higher incidence of all types of fractures in the sarcopenic subjects compared with the non-sarcopenic subjects only when the sarcopenic subjects also presented with low BMD. The HRs varied from 3.75 (95% CI 2.64–5.32) for men to 2.8 (95% CI 1.72–4.58) for women in the crude model and from 3.79 (95% CI 2.65–5.41) for men and 2.27 (95% CI 1.37–3.76) for women in the multivariable adjusted model. The results followed the same trend when traumatic fractures were excluded from the analyses. The second study[32], which had also an NOS score of 7/9, followed 5934 ambulatory community-dwelling men to determine the incidence of hip fracture and did not report any association between sarcopenia and the incidence of hip fractures (OR adjusted for age and BMD 1.17 (95% CI 0.71–1.93)).

### Length of hospital stay

Two studies[27,34] followed sarcopenic subjects to assess the impact of sarcopenia on the length of stay during hospitalization. The first study[27], which had an NOS score of 6/9, included 99 hospitalized elderly men and women aged 84.6±6.6 years. The authors did not report a significant difference in the length of hospital stay in a referral acute care unit between the sarcopenic patients (19.5 ± 16.3 days) and the non-sarcopenic patients (15.0 ± 9.9 days; p = 0.179). In contrast, the second study[34], which had an NOS score of 7/9, followed 3999 community-dwelling elderly men and women for 7 years and found a significantly higher percentage of sarcopenic men than non-sarcopenic men had a hospital stay longer than 20 days during follow-up. An adjusted OR (for age, education, COPD, diabetes mellitus, hypertension, heart disease, current smoker, MMSE, and depression) of 1.84 (95%CI 1.32–2.58) was found. No such difference was found for women.

### Hospitalization

Only one study[25], with a score of 8/9 on the NOS scale, followed sarcopenic subjects to assess the impact of sarcopenia on the incidence of hospitalization. A total of 538 community-dwelling elderly subjects aged 77.1±5.5 years were followed for a median of 55 months. Among the sarcopenic subjects (there was a 10.2% prevalence of sarcopenia), 60% were hospitalized during the follow-up versus 48% of the non-sarcopenic subjects. The risk of hospitalization was higher in sarcopenic subjects, with a crude HR of 1.57 (95% CI 1.09–2.26) and a fully adjusted HR (adjusted for age, gender, comorbidities, BMI, education, and hemoglobin) of 1.57 (95% CI 1.03–2.41).

## Discussion

The purpose of this systematic review and meta-analysis was to present and evaluate the clinical and socio-economic consequences of sarcopenia. A clear synthesis of the outcomes of sarcopenia was lacking in scientific literature. To avoid confounding consequences that were only related to low muscle mass or low muscle function separately with consequences that were directly attributable to sarcopenia itself, which is now defined by both reduced muscle mass and limited muscle function, we decided to focus on definitions that included both of these concepts. However, since the various operational definitions, proposed in the scientific literature could have different abilities to predict an outcome, only one definition was included in this systematic review (i.e., the EWGSOP definition). We decided to choose this definition over other ones because it has been largely used since its publication although it has not yet been validated as predictive of clinical outcomes. We acknowledge that this definition is presenting some weaknesses, particularly in regard to the cut-offs proposed to define low muscle mass, low muscle strength and low physical performance but, despite the absence of a worldwide consensual definition, we need to use currently available data. This systematic review could therefore provide scientific data to scientists aiming to develop one unique operational definition of sarcopenia.

No fewer than 17 prospective studies were included in our systematic review and meta-analysis. Across these studies, we identified 6 different types of outcomes. The most studied consequence of sarcopenia is mortality. Indeed, 12 studies reported data for mortality, and 10 suggested a significant relationship between sarcopenia and mortality. Because of the high number of studies focusing on this outcome, we were able to perform a meta-analysis, which indicated that sarcopenia patients faces a 4 times higher risk of mortality than non-sarcopenic subjects. The results did not vary according to the settings of the participants (community dwelling versus hospitalized subjects versus nursing home residents) or to the length of follow-up. Only age seems to have an impact on the results; as expected, there was a higher association of mortality with sarcopenia among subjects aged 79 years or older. Recently, another meta-analysis[37] that aimed to assess the association between sarcopenia and mortality was published; however, the authors did not focus on a unique definition and therefore also included studies that used only muscle mass-based definitions of sarcopenia. Nevertheless, they also found a significantly higher risk of mortality in sarcopenic subjects compared with non-sarcopenic subjects, with an HR of 1.87 (95% CI 1.61–2.18). It must be pointed out that no difference has been observed regarding the definition used for sarcopenia; a higher risk of mortality was found for sarcopenic subjects regardless of the definition used for the diagnosis. Another well-studied outcome of sarcopenia across the scientific literature is functional decline. Six out of 7 studies reporting functional decline as an outcome of sarcopenia showed a significant association. It has been suggested that sarcopenic subjects have a 3 times higher risk of functional decline or functional disability compared with non-sarcopenic subjects. Significant heterogeneity was found in this meta-analysis, probably because of the different methods used to measure functional decline (the ADL-Katz scale[25,29,33], the IAD-Lawton scale[25,29,33], the Barthel Index[27] and self-reported functional limitations[32,34]). This heterogeneity was presumed and, for this reason, we decided to use a random effects model and to perform some subgroup analyses. It should be noted that neither the age of the participants, the length of follow-up nor the tool used to measure muscle mass seemed to interact with the observed association between sarcopenia and functional decline. Four other types of consequences (i.e., the incidence of falls, the incidence of hospitalization, the incidence of fractures and the length of hospital stay) were also identified across the 17 included studies. However, the limited number of studies reporting these outcomes did not allow us to perform meta-analyses. We did not find any other reported consequences of sarcopenia in the literature based on our search strategy. It is regrettable that there are still no available regarding the consequences of sarcopenia, as defined by the EWGSOP, on quality of life. Some transversal data are available[38–41], but we did not identify any prospective studies on this topic. However, this lack is probably because, before last year, no specific quality of life questionnaire for sarcopenia was available in the literature. In 2015, a specific health-related quality of life questionnaire for sarcopenia was developed and validated by our team[42]. It should be very interesting to obtain prospective data about quality of life and its impact on individuals with sarcopenia.

Several operational definitions of sarcopenia are currently proposed in the scientific literature. Although the definition proposed by the EWGSOP is one of the most widely used in current epidemiological studies, it still needs to obtain scientific validation and be recognized as able to predict the health and clinical outcomes of sarcopenia. The present systematic review provides key elements favorable to this validation. Indeed, the majority of studies identified by this systematic review showed an association between sarcopenia, as defined by the EWGSOP, and health-related clinical outcomes.

With the exception of mortality and functional decline, for which we have a substantial number of scientific papers, there are few epidemiological studies assessing the association with other outcomes. However, our systematic review draws on the state of the art and opens doors for the development of future prospective studies. For the development of these future studies, it is important to follow some standardization regarding the definition of sarcopenia used for the diagnosis. Indeed, some studies suggest that the use of different definitions of sarcopenia has a substantial impact on its reported prevalence and outcomes[18,43]. However, it should be noted that even if the tools used to define sarcopenia have been suggested to have an important impact on the prevalence of sarcopenia[44,45], the results of our meta-analysis suggest that the impact on health-related outcomes is more limited.

This study was the first to present a list of the consequences of sarcopenia based on a systematic review. We searched in multiple electronic databases to identify a maximum possible number of studies that would meet our inclusion criteria. An important strength to highlight is that we contacted several authors of studies to obtain the data needed to compute ORs and information that was missing from the published papers. We obtained replies from 6 authors, which allowed us to include these studies in the meta-analysis. Nevertheless, this study has some limitations, particularly in the quantitative synthesis of results. Indeed, because some heterogeneity was found in the way that results were reported across studies (i.e., some authors reported HRs, some crude and some adjusted on confounding parameters, while others authors reported ORs, some crude and some adjusted), we decide to use ORs because we were able to compute ORs using the incidence data available in the papers. With this method, however, we most often reported crude ORs, which did not take into account some potential confounding factors. Moreover, there was considerable variation in the length of follow-up across studies, which can also have an impact on the results. The shortest length of follow-up was three months, while the longest was 9.8 years, which can influence the accuracy for estimating the risk of mortality or functional decline. However, we tried to take this parameter into account by performing subgroup analyses. The results did not show any effect on the length of follow-up, mortality, or functional decline. We also deplore that we were unable to assess the longitudinal loss of muscle mass and muscle strength over time. Indeed, in individual studies, muscle mass and muscle strength were only assessed at baseline to diagnose sarcopenia and these measurements were not reported over time. Reproducing these assessments over time could be raised as a perspective for further studies with a “dynamic” approach of sarcopenia. Finally, even if a large number of studies were sponsored or funded by a local foundation, national ministry, grant or national institute of research, we could not establish any relationship between the funding or a potential conflict of interest and the results of these individual studies. Notwithstanding the aforementioned limiting factors of this research, we believe that these findings can serve as a worthy reference for researchers and clinicians in their future evaluation of sarcopenia. Given its consequences, sarcopenia can be considered an important public health problem, and preventive and therapeutic interventions deserve to be further developed. The results can also serve the industry by defining an outcome point for clinical studies and assessing sample sizes for clinical trials. Furthermore, they can serve as a basis for future decision making regarding the health care system.

## Supporting Information

S1 Table PRISMA Checklist. (DOC)

## References

[1] Deschenes MR. Effects of aging on muscle fibre type and size. Sports Med. 2004;34: 809–24. 15462613 10.2165/00007256-200434120-00002

[2] Rosenberg IH. Sarcopenia: origins and clinical relevance. J Nutr. 1997/05/01. 1997;127: 990S–991S. 9164280 10.1093/jn/127.5.990S

[3] Mitchell WK, Williams J, Atherton P, Larvin M, Lund J, Narici M. Sarcopenia, Dynapenia, and the Impact of Advancing Age on Human Skeletal Muscle Size and Strength; a Quantitative Review. Front Physiol. 2012;3. 10.3389/fphys.2012.00260 PMC3429036 22934016

[4] Delmonico MJ, Harris TB, Visser M, Park SW, Conroy MB, Velasquez-Mieyer P, et al Longitudinal study of muscle strength, quality, and adipose tissue infiltration. Am J Clin Nutr. 2009;90: 1579–85. 10.3945/ajcn.2009.28047 19864405 PMC2777469

[5] Manini TM, Clark BC. Dynapenia and aging: an update. J Gerontol A Biol Sci Med Sci. 2012;67: 28–40. 10.1093/gerona/glr010 21444359 PMC3260480

[6] Clark BC, Manini TM. Functional consequences of sarcopenia and dynapenia in the elderly. Curr Opin Clin Nutr Metab Care. 2010;13: 271–6. 10.1097/MCO.0b013e328337819e 20154609 PMC2895460

[7] Muscaritoli M, Anker SD, Argilés J, Aversa Z, Bauer JM, Biolo G, et al Consensus definition of sarcopenia, cachexia and pre-cachexia: joint document elaborated by Special Interest Groups (SIG) “cachexia-anorexia in chronic wasting diseases” and “nutrition in geriatrics”. Clin Nutr. 2010;29: 154–9. 10.1016/j.clnu.2009.12.004 20060626

[8] Cruz-Jentoft AJ, Baeyens JP, Bauer JM, Boirie Y, Cederholm T, Landi F, et al Sarcopenia: European consensus on definition and diagnosis: Report of the European Working Group on Sarcopenia in Older People. Age Ageing. 2010/04/16. 2010;39: 412–423. 10.1093/ageing/afq034 20392703 PMC2886201

[9] Fielding RA, Vellas B, Evans WJ, Bhasin S, Morley JE, Newman AB, et al Sarcopenia: an undiagnosed condition in older adults. Current consensus definition: prevalence, etiology, and consequences. International working group on sarcopenia. J Am Med Dir Assoc. 2011;12: 249–56. 10.1016/j.jamda.2011.01.003 21527165 PMC3377163

[10] Dam T-T, Peters KW, Fragala M, Cawthon PM, Harris TB, McLean R, et al An evidence-based comparison of operational criteria for the presence of sarcopenia. J Gerontol A Biol Sci Med Sci. 2014;69: 584–90. 10.1093/gerona/glu013 24737561 PMC3991139

[11] Morley JE, Abbatecola AM, Argiles JM, Baracos V, Bauer J, Bhasin S, et al Sarcopenia With Limited Mobility: An International Consensus. J Am Med Dir Assoc. 2011;12: 403–409. 10.1016/j.jamda.2011.04.014 21640657 PMC5100674

[12] Studenski SA, Peters KW, Alley DE, Cawthon PM, McLean RR, Harris TB, et al The FNIH sarcopenia project: rationale, study description, conference recommendations, and final estimates. J Gerontol A Biol Sci Med Sci. 2014;69: 547–58. 10.1093/gerona/glu010 24737557 PMC3991146

[13] Baumgartner RN, Koehler KM, Gallagher D, Romero L, Heymsfield SB, Ross RR, et al Epidemiology of sarcopenia among the elderly in New Mexico. Am J Epidemiol. 1998/04/29. 1998;147: 755–763. 9554417 10.1093/oxfordjournals.aje.a009520

[14] Cooper C, Dere W, Evans W, Kanis JA, Rizzoli R, Sayer AA, et al Frailty and sarcopenia: definitions and outcome parameters. Osteoporos Int. 2012/02/01. 2012;23: 1839–1848. 10.1007/s00198-012-1913-1 22290243

[15] Chen L-K, Liu L-K, Woo Md J, Assantachai P, Auyeung T-W, Shahrul K, et al Sarcopenia in Asia: Consensus Report of the Asian Working Group for Sarcopenia. J Am Med Dir Assoc. 2014;15: 95–101. 10.1016/j.jamda.2013.11.025 24461239

[16] Cao L, Morley JE, Rosenberg H, Morley JE, Baumgartner RN, Roubenoff R, et al Sarcopenia Is Recognized as an Independent Condition by an International Classification of Disease, Tenth Revision, Clinical Modification (ICD-10-CM) Code. J Am Med Dir Assoc. Elsevier; 2016;17: 675–677. 10.1016/j.jamda.2016.06.001 27470918

[17] Beaudart C, Rizzoli R, Bruyere O, Reginster JY, Biver E. Sarcopenia: Burden and challenges for Public Health. 2014. Archives of Public Health. Arch Public Heal. 2014;72:45. 10.1186/2049-3258-72-45 PMC4373245 25810912

[18] Bischoff-Ferrari HA, Orav JE, Kanis JA, Rizzoli R, Schlögl M, Staehelin HB, et al Comparative performance of current definitions of sarcopenia against the prospective incidence of falls among community-dwelling seniors age 65 and older. Osteoporos Int. 2015; 10.1007/s00198-015-3194-y 26068298

[19] Vetrano DL, Landi F, Volpato S, Corsonello A, Meloni E, Bernabei R, et al Association of sarcopenia with short- and long-term mortality in older adults admitted to acute care wards: results from the CRIME study. J Gerontol A Biol Sci Med Sci. 2014/04/20. 2014;69: 1154–1161. 10.1093/gerona/glu034 24744390

[20] Sánchez-Rodríguez D, Marco E, Miralles R, Fayos M, Mojal S, Alvarado M, et al Sarcopenia, physical rehabilitation and functional outcomes of patients in a subacute geriatric care unit. Arch Gerontol Geriatr. 2014;59: 39–43. 10.1016/j.archger.2014.02.009 24726179

[21] Landi F, Cruz-Jentoft AJ, Liperoti R, Russo A, Giovannini S, Tosato M, et al Sarcopenia and mortality risk in frail older persons aged 80 years and older: results from ilSIRENTE study. Age Ageing. 2013/01/17. 2013;42: 203–209. 10.1093/ageing/afs194 23321202

[22] Landi F, Liperoti R, Russo A, Giovannini S, Tosato M, Capoluongo E, et al Sarcopenia as a risk factor for falls in elderly individuals: results from the ilSIRENTE study. Clin Nutr. 2012/03/15. 2012;31: 652–658. 10.1016/j.clnu.2012.02.007 22414775

[23] Landi F, Liperoti R, Fusco D, Mastropaolo S, Quattrociocchi D, Proia A, et al Sarcopenia and mortality among older nursing home residents. J Am Med Dir Assoc. 2011/08/23. 2012;13: 121–126. 10.1016/j.jamda.2011.07.004 21856243

[24] Cerri AP, Bellelli G, Mazzone A, Pittella F, Landi F, Zambon A, et al Sarcopenia and malnutrition in acutely ill hospitalized elderly: Prevalence and outcomes. Clin Nutr. 2015;34: 745–751. 10.1016/j.clnu.2014.08.015 25263170

[25] Bianchi L, Ferrucci L, Cherubini A, Maggio M, Bandinelli S, Savino E, et al The Predictive Value of the EWGSOP Definition of Sarcopenia: Results From the InCHIANTI Study. J Gerontol A Biol Sci Med Sci. Oxford University Press; 2016;71: 259–64. 10.1093/gerona/glv129 26333772 PMC4723661

[26] Saka B, Ozkaya H, Karisik E, Akin S, Akpinar TS, Tufan F, et al Malnutrition and sarcopenia are associated with increased mortality rate in nursing home residents: A prospective study. Eur Geriatr Med. 2016;7: 232–238.

[27] Sánchez-Rodríguez D, Marco E, Miralles R, Guillén-Solà A, Vázquez-Ibar O, Escalada F, et al Does gait speed contribute to sarcopenia case-finding in a postacute rehabilitation setting? Arch Gerontol Geriatr. 2015;61: 176–181. 10.1016/j.archger.2015.05.008 26051706

[28] Alexandre T da S, Duarte YA de O, Santos JLF, Wong R, Lebrão ML. Sarcopenia according to the European Working Group on Sarcopenia in Older People (EWGSOP) versus dynapenia as a risk factor for mortality in the elderly. J Nutr Health Aging. 2014;18: 751–6. 10.1007/s12603-014-0450-3 25286455 PMC12877533

[29] da Silva Alexandre T, de Oliveira Duarte YA, Ferreira Santos JL, Wong R, Lebrao ML. Sarcopenia according to the european working group on sarcopenia in older people (EWGSOP) versus Dynapenia as a risk factor for disability in the elderly. J Nutr Heal Aging. 2014/06/03. 2014;18: 547–553. 10.1007/s12603-014-0465-9 PMC12880480 24886743

[30] Arango-Lopera VE, Arroyo P, Gutierrez-Robledo LM, Perez-Zepeda MU, Cesari M. Mortality as an adverse outcome of sarcopenia. J Nutr Heal Aging. 2013/03/06. 2013;17: 259–262. 10.1007/s12603-012-0434-0 PMC4764255 23459979

[31] Chalhoub D, Cawthon PM, Ensrud KE, Stefanick ML, Kado DM, Boudreau R, et al Risk of Nonspine Fractures in Older Adults with Sarcopenia, Low Bone Mass, or Both. J Am Geriatr Soc. 2015;63: 1733–1740. 10.1111/jgs.13605 26310882 PMC4625906

[32] Cawthon PM, Blackwell TL, Cauley J, Kado DM, Barrett-Connor E, Lee CG, et al Evaluation of the Usefulness of Consensus Definitions of Sarcopenia in Older Men: Results from the Observational Osteoporotic Fractures in Men Cohort Study. J Am Geriatr Soc. 2015;63: 2247–59. 10.1111/jgs.13788 26502831 PMC4739621

[33] Tanimoto Y, Watanabe M, Sun W, Tanimoto K, Shishikura K, Sugiura Y, et al Association of sarcopenia with functional decline in community-dwelling elderly subjects in Japan. Geriatr Gerontol Int. 2013/03/05. 2013; 10.1111/ggi.12037 23452074

[34] Woo J, Leung J, Morley JE. Defining Sarcopenia in Terms of Incident Adverse Outcomes. J Am Med Dir Assoc. 2015;16: 247–252. 10.1016/j.jamda.2014.11.013 25548028

[35] Kim JH, Lim S, Choi SH, Kim KM, Yoon JW, Kim KW, et al Sarcopenia: an independent predictor of mortality in community-dwelling older korean men. J Gerontol A Biol Sci Med Sci. 2014/04/12. 2014;69: 1244–1252. 10.1093/gerona/glu050 24721723

[36] Kim JH, Lim S, Choi SH, Kim KM, Yoon JW, Kim KW, et al Sarcopenia: an independent predictor of mortality in community-dwelling older Korean men. J Gerontol A Biol Sci Med Sci. 2014;69: 1244–52. 10.1093/gerona/glu050 24721723

[37] Chang S-F, Lin P-L. Systematic Literature Review and Meta-Analysis of the Association of Sarcopenia With Mortality. Worldviews Evid Based Nurs. 2016; 10.1111/wvn.12147 26844538

[38] Silva Neto LS, Karnikowiski MG, Tavares AB, Lima RM. Association between sarcopenia, sarcopenic obesity, muscle strength and quality of life variables in elderly women. Rev Bras Fisioter. 2012/09/18. 2012;16: 360–367. 22983215

[39] Yadav A, Chang Y-H, Carpenter S, Silva AC, Rakela J, Aqel BA, et al Relationship between sarcopenia, six-minute walk distance and health-related quality of life in liver transplant candidates. Clin Transplant. 2015;29: 134–41. 10.1111/ctr.12493 25430554

[40] Morishita S, Kaida K, Tanaka T, Itani Y, Ikegame K, Okada M, et al Prevalence of sarcopenia and relevance of body composition, physiological function, fatigue, and health-related quality of life in patients before allogeneic hematopoietic stem cell transplantation. Support Care Cancer. 2012/04/25. 2012;20: 3161–3168. 10.1007/s00520-012-1460-5 22526152

[41] Go SW, Cha YH, Lee JA, Park HS. Association between Sarcopenia, Bone Density, and Health-Related Quality of Life in Korean Men. Korean J Fam Med. 2013/08/02. 2013;34: 281–288. 10.4082/kjfm.2013.34.4.281 23904958 PMC3726796

[42] Beaudart C, Biver E, Reginster J-Y, Rizzoli R, Rolland Y, Bautmans I, et al Development of a self-administrated quality of life questionnaire for sarcopenia in elderly subjects: the SarQoL. Age Ageing. Oxford University Press; 2015;44: 960–966. 10.1093/ageing/afv133 26433796 PMC4621234

[43] Dupuy C, Lauwers-Cances V, Guyonnet S, Gentil C, Abellan Van Kan G, Beauchet O, et al Searching for a relevant definition of sarcopenia: results from the cross-sectional EPIDOS study. J Cachexia Sarcopenia Muscle. 2015;6: 144–54. 10.1002/jcsm.12021 26136190 PMC4458080

[44] Beaudart C, Reginster JY, Slomian J, Buckinx F, Dardenne N, Quabron A, et al Estimation of sarcopenia prevalence using various assessment tools. Exp Gerontol. 2014/12/03. 2014;61C: 31–37. 10.1016/j.exger.2014.11.014 25449859

[45] Bijlsma AY, Meskers MC, Molendijk M, Westendorp RG, Sipila S, Stenroth L, et al Diagnostic measures for sarcopenia and bone mineral density. Osteoporos Int. 2013/05/08. 2013;24: 2681–2691. 10.1007/s00198-013-2376-8 23649802

